# A deterministic genotyping workflow reduces waste of transgenic individuals by two-thirds

**DOI:** 10.1038/s41598-021-94288-0

**Published:** 2021-07-28

**Authors:** Frederic Strobl, Ernst H. K. Stelzer

**Affiliations:** Physical Biology/Physikalische Biologie (IZN, FB 15), Buchmann Institute for Molecular Life Sciences (BMLS), Cluster of Excellence Frankfurt – Macromolecular Complexes (CEF – MC), Goethe-Universität Frankfurt Am Main (Campus Riedberg), Max-von-Laue-Straße 15, 60438 Frankfurt am Main, Germany

**Keywords:** Genotyping and haplotyping, Genetic vectors, Transgenic organisms, Entomology

## Abstract

We present a deterministic workflow for genotyping single and double transgenic individuals directly upon nascence that prevents overproduction and reduces wasted animals by two-thirds. In our vector concepts, transgenes are accompanied by two of four clearly distinguishable transformation markers that are embedded in interweaved, but incompatible Lox site pairs. Following Cre-mediated recombination, the genotypes of single and double transgenic individuals were successfully identified by specific marker combinations in 461 scorings.

## Introduction

Transgenesis of established and emerging model organisms is a core method in cell and developmental biology^1^. Since nearly all metazoan species are diploid, usual mating schemes involving one transgene result in wild-type, hemizygous and homozygous individuals. With two distinct transgenes, the complexity rises considerably, and mating schemes result in up to nine genotypes. Transformation markers indicate whether a respective transgene is present, but cannot separate hemi- from homozygosity^2^. Hence, genetic assays are required, which are usually invasive and waste resources such as time, manpower as well as consumables^3,4^.

Further, for many metazoan model organisms, the delay between progeny nascence and the time point at which genetic assays become feasible is inconveniently long. In consequence, stochastically justified overproduction is the only practicable approach to ensure that the necessary number of suitable descendants is later available for research purposes, which is always associated with a considerable waste of animals^5^. For instance, if a hemizygote is mated with a genotypically identical sibling, one out of four descendants is expected to be homozygous. On the single case level, however, and due to purely stochastic reasons, there will be occasions in which ten or even more wild types and/or hemizygotes precede the first homozygote. Consequently, far more than four descendants must be produced and maintained in order to minimize the probability having no suitable individual available at the time of experimentation. This economic as well as ethical issue can be addressed by establishing and applying deterministic workflows in which the genotype is identified directly upon nascence and production is stopped immediately once the number of suitable individuals is met.

We already established the AGameOfClones vector concept (AGOC), which provides full visual control over one transgene and thus allows the systematic creation of homozygous transgenic lines^6^. It uses mOrange-based^7^ and mCherry-based^8^ eye-specific^9^ transformation markers (mO and mC, respectively), which are clearly distinguishable. Both markers are embedded in interweaved, but incompatible Lox site pairs^10,11^. Cre-mediated recombination results in hemizygous individuals in which the transgene is accompanied by only one of both markers. In the following generation, heterozygous descendants are selected by the presence of both markers and, when mated, produce homozygous progeny that are revealed by the lack of one marker. Most importantly, since AGOC-based genotyping is non-invasive and takes only a few seconds per individual, descendants can be assayed directly after nascence.

Our AClashOfStrings vector concept (ACOS) is a functionally identical but color-shifted variant of AGOC. Instead of mO and mC, mCerulean-based^12^ and mVenus-based^13^ transformation markers (mCe and mVe, respectively) are used. Using appropriate filter sets, all four markers are clearly distinguishable. Similar to AGOC, ACOS provides full visual control over one transgene, while the combined AGameOfClones/AClashOfStrings vector concept (AGOC/ACOS) now provides control over two distinct transgenes.

## Results

Our proof-of-principle for ACOS and AGOC/ACOS relied on the red flour beetle *Tribolium castaneum*, an emerging insect model organism^14,15^, and covered the systematic creation of single and double homozygous transgenic lines. At first, we created a transformation-ready ACOS vector that mediates expression of mRuby-labeled *histone H2B* under control of the *tubulin alpha 1-like protein* promoter (Figure S1). Injection of 250 embryos resulted in 27 fertile F1 adults (10.8%), which were mated with wild types. Screening of the F2 progeny revealed three independent germline transformation events (11.1%), which is roughly on par with previously published ratios^16^. Consequently, three F2 founder females, one for each transformation event, were mated with wild-type males to establish three independent sublines called ACOS{ATub’H2B-mRuby} #1 to #3. The subsequent mating procedure for the systematic creation of single homozygous transgenic lines spanned four generations (Figure S2) and was successfully completed for all three sublines, which eventually resulted in F7 (mCe/mCe) and (mVe/mVe) homozygotes (Table S1).

**Figure 1:**
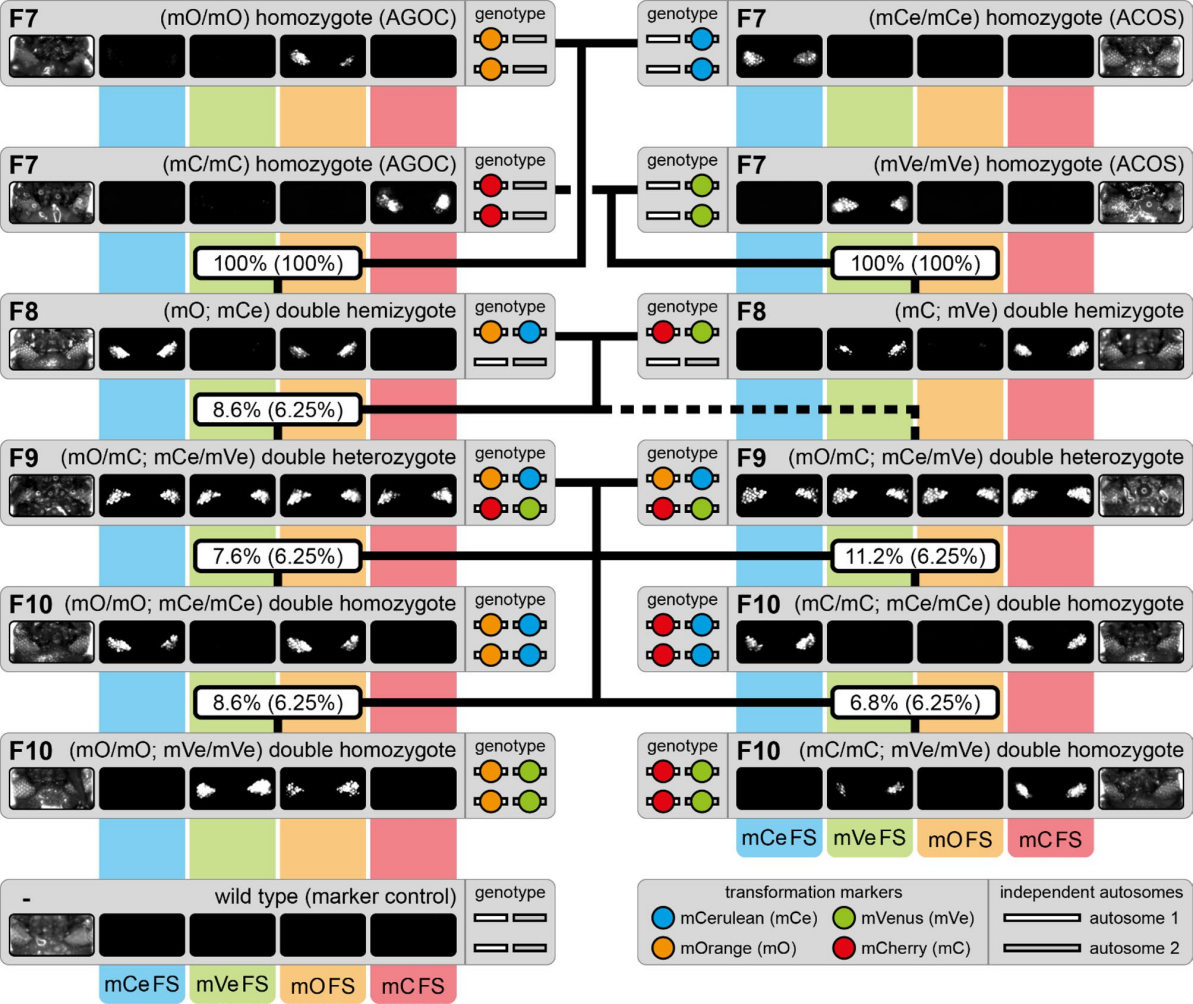
The AGOC/ACOS-associated F7 to F10 mating procedure for the systematic creation of double homozygous transgenic lines demonstrated for the Gruul #1 subline. Gray rectangles show the phenotypes and illustrate the genotypes for two independent autosomes. White and gray bars represent the AGOC and ACOS transgene locations, respectively. From the F7 to the F10 generation, the genotypes were phenotypically determined by monitoring mO, mC, mCe and mVe. The percentage boxes indicate the experimental (and theoretical) ratios of the progeny that showed the respective phenotype. The dashed line represents genotypically identical siblings. FS, filter set.

For the systematic creation of double homozygous lines, all three sublines were hybridized with the AGOC{Zen1’#O(LA)-mEmerald} #1 subline^6^. For convenience, the hybrid sublines were named Gruul #1 to #3. The mating procedure spanned three more generations and was phenotypically documented for the Gruul #1 subline (Fig. 1):

1. F7 (mO/mO) homozygous females (AGOC), which carried only mO on both the maternal and paternal chromosome, were mated with F7 (mCe/mCe) homozygous males (ACOS), which carried only mCe on both the maternal and paternal chromosome (Fig. 1, upper ‘F7’ rectangles). In parallel, F7 (mC/mC) homozygous females (AGOC), which carried only mC on both the maternal and paternal chromosome, were mated with F7 (mVe/mVe) homozygous males (ACOS), which carried only mVe on both the maternal and paternal chromosome (Fig. 1, lower ‘F7’ rectangles). Both crosses resulted in 100% F8 (mO; mCe) and 100% F8 (mC; mVe) double hemizygotes, respectively.
2. F8 (mO; mCe) double hemizygous females were mated with F8 (mC; mVe) double hemizygous males (Fig. 1, ‘F8’ rectangles), which resulted in F9 (mO/mC; mCe/mVe) double heterozygotes that carried all four markers (Table 1, ‘F8’ row). This was demonstrated by mating respective F9 double heterozygous females with wild-type males and scoring the progeny (Table S2, ‘F9-S’ row).
3. F9 (mO/mC; mCe/mVe) double heterozygous females were mated with genotypically identical F9 male siblings (Fig. 1, ‘F9’ rectangles), which resulted in F10 (mO/mO; mCe/mCe), (mC/mC; mCe/mCe), (mO/mO; mVe/mVe) and (mC/mC; mVe/mVe) double homozygotes (Fig. 1, ‘F10’ rectangles and Table 1, ‘F9’ row). This was demonstrated by mating respective F10 double homozygous females with wild-type males and scoring the progeny (Table S2, ‘F10’ rows).

**Table 1:**
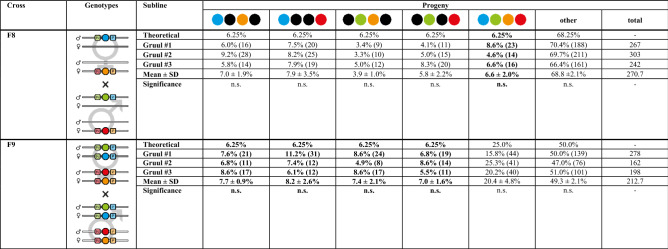
Mating procedure results for the Gruul #1 to #3 hybrid sublines from the F8 to the F10 generation.

Both F7 crosses (cf. Figure 1) resulted in 100% F8 (mO; mCe) and 100% F8 (mC; mVe) double hemizygotes, respectively. Progeny that were used in the subsequent crosses or to establish the F10+ continuative cultures are marked bold. The numbers in brackets and in the ‘total’ sub-column indicate the number of scored individuals. One-sample/two-tailed Student’s t-tests revealed no significant differences (n.s.) between the arithmetic means and the theoretical Mendelian ratios.

Throughout all generations, the progeny scores matched the expectations. No significant differences between the respective arithmetic means and the theoretical Mendelian ratios were found. Importantly, all expected phenotypes, and thus all expected genotypes, were found in all generations and consequently, F10 double homozygotes with all four possible marker combinations could be obtained for all three Gruul sublines.

**Figure 2:**
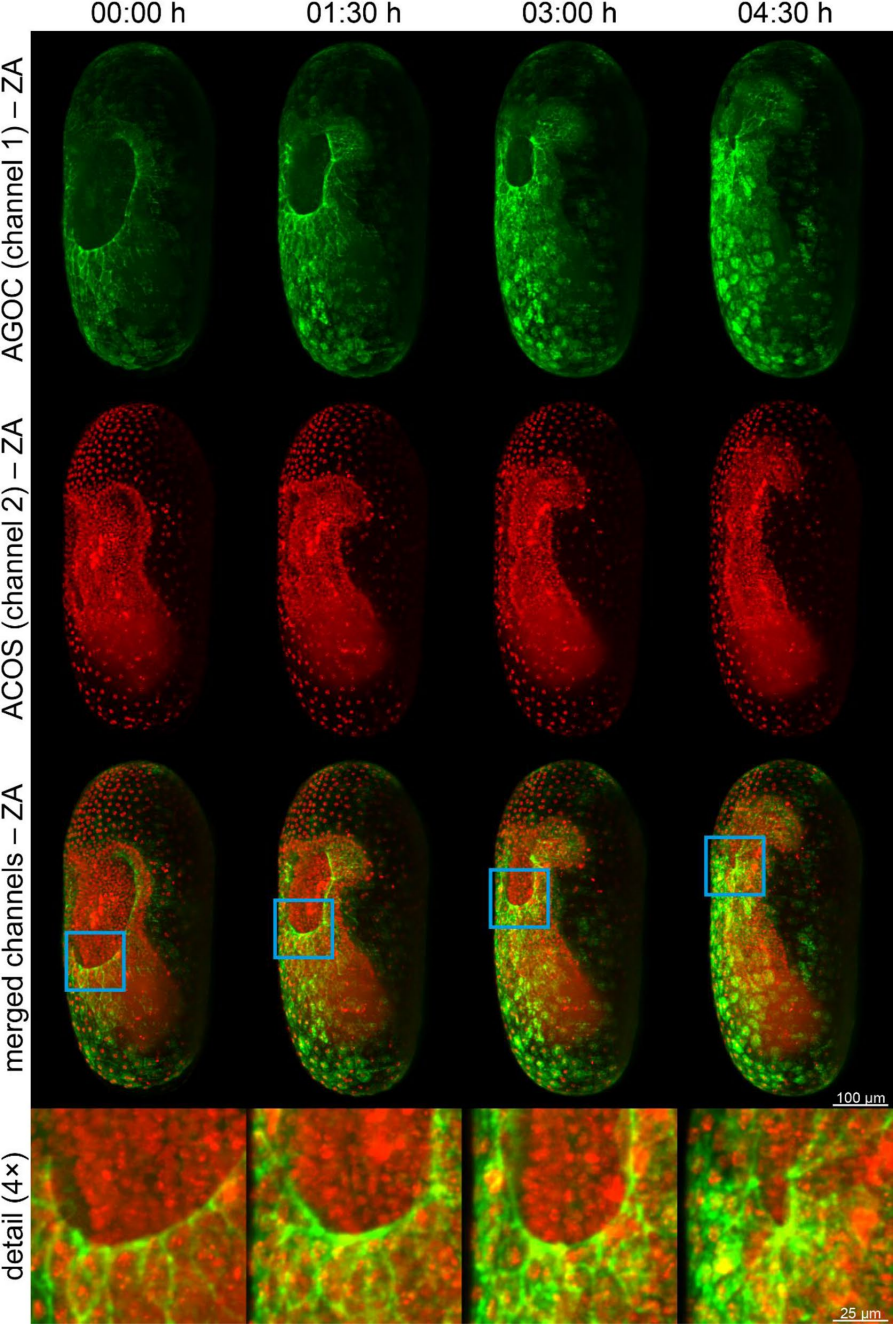
Fluorescence live imaging of a (mO/mO; mCe/mCe) double homozygous *Tribolium* embryo from the Gruul #1 hybrid subline during gastrulation. The tissue-specific expression of mEmerald-labeled Lifeact (green, first row) visualizes the actin cytoskeleton in the extra-embryonic serosa cells, while mRuby-labeled *histone H2B* (red, second row) visualizes cell nuclei. This hybrid subline provides essential data for investigations of the complex morphogenetic dynamics of embryonic and extra-embryonic tissues during serosa window closure (third row and detail images). ZA, Z maximum projection with intensity adjustment.

The AGOC- and ACOS-associated vectors are primarily designed to create transgenic lines suitable for fluorescence live imaging. Thus, we imaged embryos from the Gruul #1 subline using digitally scanned laser light sheet-based fluorescence microscopy (DSLM)^17,18^ relying on previously published protocols for *Tribolium*^19,20^. This hybrid subline shows adequate signal to visualize the complex morphogenetic dynamics of the serosa, one of the two extra-embryonic membranes in *Tribolium*, during gastrulation (Fig. 2, Movie 1).

Lastly, we assayed reversibility and repeatability, multimodality as well as long-term robustness of our vector concepts. To resegregate the transgenes, F11 (mO; mCe) as well as F11 (mC; mVe) double hemizygous females (cf. Table S2) of all three Gruul sublines were mated with wild-type males, which resulted in F12 (mO) and (mC) hemizygotes (AGOC) as well as F12 (mCe) and (mVe) hemizygotes (ACOS). These descendants, which carried only one of the transgenes, were used to repeat the mating procedure for the systematic creation of double homozygous lines with alternative marker combinations:

1. F12 (mO) hemizygous females were mated with F12 (mVe) hemizygous males, while in parallel, F12 (mC) hemizygous females were mated with F12 (mCe) hemizygous males, which resulted in F13 (mO; mVe) and (mC; mCe) double hemizygotes, respectively (Table 2, ‘F12-Sh’ and ‘F12-Lo’ rows). Due to the alternative parental composition, these descendants carried a different marker combination than their corresponding F8 ancestors.
2. F13 (mO; mVe) double hemizygous females were mated with F13 (mC; mVe) double hemizygous males, which resulted in F14 progeny that were genotypically identical to their corresponding F9 ancestors (Table 2, ‘F13’ row) as demonstrated by mating respective females with wild-type males (Table S3, ‘F14-S’ row).
3. F14 (mO/mC; mCe/mVe) double heterozygous females were mated with genotypically identical F14 male siblings, which resulted in F15 progeny that were genotypically identical to their corresponding F10 ancestors (Table 2, ‘F14’ row) as demonstrated by mating respective females with wild-type males (Table S3, ‘F15’ rows).

**Table 2:**
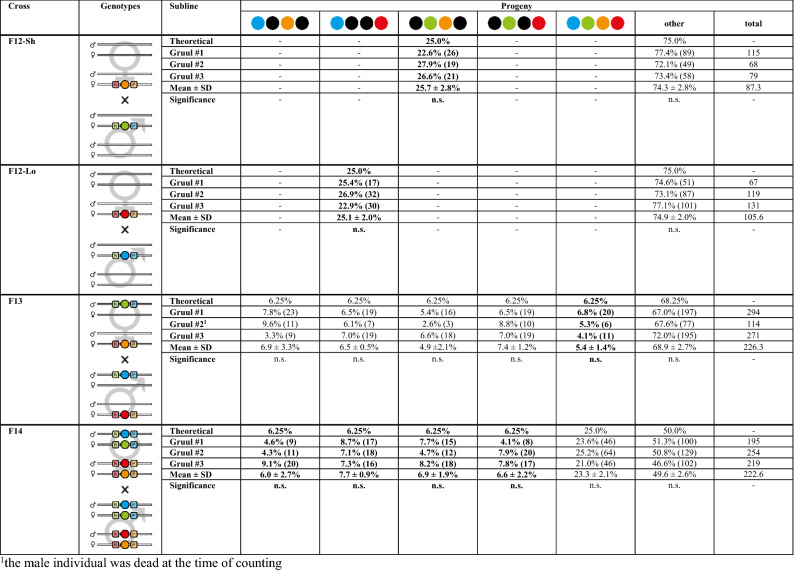
Mating procedure results for the Gruul #1 to #3 hybrid sublines from the F12 to the F15 generation.

Progeny that were used in the subsequent crosses are marked bold. The numbers in brackets and in the ‘total’ sub-column indicate the number of scored individuals. One-sample/two-tailed Student’s t-tests revealed no significant differences (n.s.) between the arithmetic means and the theoretical Mendelian ratios.

^1^The male parent was dead at the time of counting.

Similar to the F7 to F10 mating procedure results, all expected phenotypes and genotypes were found in all generations. Consequently, F15 double homozygotes, which were genotypically identical to their corresponding F10 ancestors, could be obtained for all three Gruul sublines.

## Discussion

In our study, we demonstrated the functionality of ACOS and AGOC/ACOS, two vector concepts that provide full visual control over one or two transgenes, respectively. In the single transgene scenario, ACOS performs as reliably as AGOC^6^ and provides the same benefits. In brief, it saves resources, supports unsynchronized genotyping of single individuals and functions even if the insertion junction is unknown, as is the case for the ACOS{ATub’H2B-mRuby} #3 subline. Further, in a total of 172 scorings, the progeny marker distribution always confirmed the parental genotypes (Table S4, upper row), which indicates that ACOS is error resistant. Accordingly, AGOC/ACOS provides all these benefits in the considerably more complex double transgene scenario. Unsynchronized genotyping of single individuals is more often necessary when two distinct transgenes are involved, and the approach also functions if both insertion junctions are unknown. Further, during the mating procedure for the systematic creation of double homozygous lines, in a total of 289 scorings, the progeny marker distribution always confirmed the parental genotypes (Table S4, middle and lower row). This suggests that our approach performs reliably even with two transgenes, which is especially important for experiments in which a single partially incorrectly genotyped individual threatens the integrity of results and conclusions.

However, our concept is also subject to several limitations. Firstly, fluorescence-based transformation markers may impair the respective spectral range for further use. Secondly, even though our concepts do not require consumables, the initial investments to acquire suitable fluorescence filter sets are rather high. Both of these limitations can be addressed by using eye/cuticle pigmentation-based markers, which have the additional advantage that scoring can be performed even without the aid of a microscope^21,22^. Thirdly, a second transformation marker increases transgene size, in our case by 1,241 bp (26.3%). For piggyBac-based germline transformation in *Tribolium*, it was shown that larger transgenes are associated with slightly lower transformation rates^16^. Lastly, the arguably most inconvenient limitation is that our concepts cannot be applied retroactively, but have to be considered already during the planning phases of experiments involving transgenesis.

In our study, we used AGOC/ACOS to combine sublines primarily designed for fluorescence live imaging into two-colored hybrid sublines to visualize the actin cytoskeleton and cell nuclei in parallel. Such two-color sublines can also be based on internal ribosomal entry sites^23^ or the T2A peptide^24^, which requires less overall effort. However, such an approach lacks the modular character of AGOC/ACOS, which allows to combine proven AGOC and ACOS sublines with relatively little effort. Abstractly seen, AGOC/ACOS has a certain similarity to the GAL4/UAS system^25^, but operates at a higher functional level. While the latter is intended for modular combination of promoters and coding sequences to form ‘binary expression cassettes’, our concept allows modular combination of two independent expression cassettes.

With our vector concepts, overproduction is almost completely prevented. The production of descendants with suitable genotypes is equivalent to a Bernoulli process. However, when genotypes are determined by using genetic assays, the delay between progeny nascence and the time point where genetic assays become feasible necessitates a stochastic workflow. A complementary cumulative binomial distribution function can be used to estimate the probability of having the required number of suitable individuals available at the time of experimentation. This probability depends on the number of descendants that are initially produced in conjunction with the Mendelian probability to inherit the suitable genotype. During the respective mating procedures for the systematic creation of single and double homozygous transgenic lines, the Mendelian probability to obtain suitable F6 and F9/F14 descendants equals 25% respectively 6.25% (cf. Figure S2 and Fig. 1). In stochastic workflows, at least 11 respectively 47 descendants must be initially produced to achieve a probability higher than 95% to obtain at least one suitable individual. In contrast, our vector concepts allow a deterministic workflow. By identifying the genotype directly upon nascence, further production can be stopped once a suitable individual is identified, which occurs on average after 4 respectively 16 descendants. Hence, our approach reduces the required number of progeny by about two-thirds, since the obligatory ‘safety margins’ associated with stochastic workflows are no longer necessary. In absolute terms, the number of wasted animals decreases exponentially with the number of distinct transgenes.

We demonstrated the functionality of our approach in *Tribolium*, however our concept is easily adapted for other diploid species. The vectors are directly applicable in many insect model organisms, including *Drosophila melanogaster*^9^, and require only minor alteration for non-insect species since they were designed with modularity in mind^6^. Especially for the work with vertebrate model organisms, which are under special legal protection in many countries^26,27^, our vector concept is particularly attractive. For instance, many zebrafish laboratories still use the fin-clipping approach for genotyping, primarily on adult zebrafish^28^. With our concepts and suitable transformation markers^29^, selection is already possible at the embryonic stage, which is for example specifically exempted in the animal welfare legislation of the European Union^30,31^. Regarding mouse as a mammalian species, the situation is slightly more complex and therefore best explained by means of an example. If a certain number of embryos with specific genotypes should be collected, our concepts allow to sacrifice pregnant mothers one-by-one until the required number is reached, as assessment takes only several seconds per embryo. In contrast, the incubation periods in the fastest genetic assay-based genotyping protocol that we are aware of requires about one hour^32^, which conflicts with the abovementioned one-by-one strategy. However, possible markers and their combination capabilities should be thoroughly verified prior to adaptation^33–36^. Taken together, future adaptations of our concept to commonly used vertebrate model organisms have a high potential to strengthen the ethically motivated animal welfare endeavor in accordance with the 3R principles^37^.

## Methods

### Model organism

In this study, the *Tribolium castaneum* (red flour beetle, NCBITaxon:7070) PWAS^6^ background strain was used, which carries the pearl^38^ and light ocular diaphragm mutations^39^. All animal-related experiments were approved by the Goethe University institutional ethics committee (Tierschutzkommission der Goethe-Universität, file number TSB20210616_BB) and performed in agreement with the German Animal Welfare Act (Tierschutzgesetz/Tierschutz-Versuchstierordnung, based on ETS No.123^30^ and EU Directive 2010/63/EU^31^) as well as the ARRIVE 2.0 guidelines^40^.

### Molecular biology: the pACOS{ATub’H2B-mRuby} vector

An artificial sequence, consisting of (i) an AvrII restriction enzyme site, (ii) a LoxP site, (iii) the mCerulean-based transformation marker (mCe) expression cassette, (iv) a LoxN site, (v) a LoxP site, (vi) the mVenus-based transformation maker (mVe) expression cassette, (vii) a LoxN site and (viii) a XhoI restriction enzyme site, was excised from pGS[ACOS]^6^ with AvrII and XhoI and inserted (in reverse orientation) into slots #3 and #4 of the accordingly digested pAGOC{#P’#O(LA)-mEmerald} vector^6^, replacing the initial mO and mC expression cassettes. The resulting vector was termed pACOS{#P’#O(LA)-mEmerald} and used as an intermediate vector for further cloning operations.

A second artificial sequence, consisting of (i) a FseI restriction enzyme site, (ii) a two-slot cloning site that is composed of a promoter (#P) and an open-reading frame (#O) slot that carries the human-derived GAP43 membrane anchor tag by default^41^, (iii) a 9 bp Ala-Ala-Ala linker, (iv) the codon-optimized mRuby2^42^ open-reading frame and (v) a SbfI restriction enzyme, was de novo synthesized and inserted into the unique SacI and KpnI sites of pMA-T (Thermo Fisher Scientific). The resulting vector was termed pGS[#P’#O(MEM)-mRuby]. The insert was excised from the backbone with FseI/SbfI and inserted into the accordingly digested pACOS{#P’#O(LA)-mEmerald} vector, replacing the *Saccharomyces cerevisiae*-derived Lifeact peptide tag and mEmerald open-reading frames. The resulting vector was termed pACOS{#P’#O(MEM)-mRuby} and used as an intermediate vector for further cloning operations.

A recombinant sequence, consisting of the *Tribolium castaneum*-derived *tubulin alpha 1-like protein* promoter and the *Tribolium castaneum*-derived *histone H2B* open-reading frame, was excised from pTC-ATub’H2B-GEM-T Easy^6^ with AscI and NotI and inserted into #P’#O of the accordingly digested pACOS{#P’#O(MEM)-mRuby} vector. The resulting vector was termed pACOS{ATub’H2B-mRuby} and used for germline transformation.

### Molecular biology: the pFIRE{HSP68’NLS-Cre} vector

The mCherry-based eye-specific transformation marker (mC) expression cassette, consisting of (i) the artificial 3×P3 promoter, (ii) the codon-optimized open-reading frame for mCherry^8^ and (iii) the SV40 poly(A), was PCR amplified from pAGOC^6^ by using the 5’-AAATTT**CCTAGG**GTTCCCACAATGGTTAATTCGAG-3’ and 5’-AAATTT**GCTAGC**GTTTAAACCTTAAGTAAGATACATTGATGAGTTTGG-3’ overhang primers, which introduced upstream an AvrII and downstream a NheI site (overhang underlined, restriction enzyme sites in bold). The PCR product and the pICE{HSP68’NLS-Cre} vector were digested accordingly to replace the initial mCe with the mC expression cassette. The resulting vector was termed pFIRE{HSP68’NLS-Cre} and used for germline transformation.

### Germline transformation, crossing setups and insertion junction sequencing

Germline transformation with both transformation-ready vectors and establishment of the FIRE{HSP68’NLS-Cre} #1 helper subline (following the example of the ICE{HSP68’NLS-Cre} #1 helper subline) was performed as described before^6^. For each crossing step, up to six female-male pairs were mated in small glass vials filled with 1.5 g, 2.5 g (F4 cross) or 3.5 g (F8, F9, F14 and F15 crosses) of growth medium^6^. Progeny were scored for transformation marker presence during either the larval, pupal and adult stage by using a fluorescence stereo microscope (SteREO Discovery.V8, Zeiss) with appropriate filter sets (Table S5). Using previously published protocols and primers^6^, the insertion junctions for the ACOS{ATub’H2B-mRuby} #1 and #2 as well as the FIRE{HSP68’NLS-Cre} #1 sublines were successfully determined (Tcas5.2 genome assembly^43^, TTAA sites at ChLG6:7916392-5, ChLG7:14927754-7 and ChLGX: 2014273-6, respectively). A one-sample/two-tailed Student’s t-test was performed to determine whether the arithmetic means differ significantly from the theoretical Mendelian ratios.

### Light sheet-based fluorescence microscopy

Long-term live imaging was performed with digitally scanned laser light sheet-based fluorescence microscopy (DSLM, LSFM) as described previously^20,44^. In brief, embryo collection was performed with (i) the F7+ continuative (mO/mO) homozygous AGOC{Zen1’#O(LA)-mEmerald} #1 subline (dataset DS0001), (ii) the F7+ continuative (mCe/mCe) homozygous ACOS{ATub’H2B-mRuby} #1 (dataset DS0002) and (iii) the F10+ continuative (mO/mO; mCe/mCe) double homozygous Gruul #1 subline (dataset DS0003) for 1 h at 25 °C, and embryos were incubated for 15 h at 25 °C. Sample preparation took approximately 1 h at room temperature (23 ± 1 °C), so that embryos were at the onset of gastrulation. Embryos were recorded along four pair-wise orthogonal directions, that is, along the orientations 0°, 90°, 180° and 270°, with a time interval of 30 min. All shown embryos survived the imaging procedure, developed into healthy and fertile adults, and when mated with wild types, produced only transgenic progeny that were also fertile. Metadata for the three datasets are provided in Table S6.

## Supplementary Information

Supplementary Figure S1.

Supplementary Figure S2.

Supplementary Table S1.

Supplementary Table S2.

Supplementary Table S3.

Supplementary Table S4.

Supplementary Table S5.

Supplementary Table S6.

Supplementary Video 1.

Supplementary Legends.

## Data Availability

All non-microscopy data is provided within the manuscript and the supplementary data. Microscopy data can be accessed as described in Table S6.
