# Author Correction: A deterministic genotyping workflow reduces waste of transgenic individuals by two-thirds

**DOI:** 10.1038/s41598-021-99823-7

**Published:** 2021-10-07

**Authors:** Frederic Strobl, Ernst H. K. Stelzer

**Affiliations:** Physical Biology/Physikalische Biologie (IZN, FB 15), Buchmann Institute for Molecular Life Sciences (BMLS), Cluster of Excellence Frankfurt – Macromolecular Complexes (CEF – MC), Goethe-Universität Frankfurt Am Main (Campus Riedberg), Max-von-Laue-Straße 15, 60438 Frankfurt am Main, Germany

Correction to: *Scientific Reports* 10.1038/s41598-021-94288-0, published online 28 July 2021

The original version of this Article contained errors where Table S5 and Table S6 were incorrectly cited. As the result, in the Methods section, under the subheading ‘Germline transformation, crossing setups and insertion junction sequencing’,

“Progeny were scored for transformation marker presence during either the larval, pupal and adult stage by using a fluorescence stereo microscope (SteREO Discovery.V8, Zeiss) with appropriate filter sets (Table S4).”

now reads:

“Progeny were scored for transformation marker presence during either the larval, pupal and adult stage by using a fluorescence stereo microscope (SteREO Discovery.V8, Zeiss) with appropriate filter sets (Table S5).”

And, under the subheading ‘Light sheet-based fluorescence microscopy’,

“Metadata for the three datasets are provided in Table S5.”

now reads:

“Metadata for the three datasets are provided in Table S6.”

In Data availability section,

“Microscopy data can be accessed as described in Table S5.”

now reads:

“Microscopy data can be accessed as described in Table S6.”

Additionally, in the Supplementary Information 8 file, the “Data Access” row was omitted in Table S6. The “Data Access” row now reads:

**Table:** 

*Dataset (DS)*	DS0001	DS0002	DS0003
*Dataset Access*	DOI: 10.5281/zenodo.4892363	DOI: 10.5281/zenodo.4892373	DOI: 10.5281/zenodo.4892381

The original Supplementary Information 8 file is provided below.

Finally, the Supplementary Information 1 and 5 files published with this Article contained tracked changes, these have now been removed.

The original Article and accompanying Supplementary Information files have been corrected.

## Supplementary Information

Supplementary Information.

