# Supplementary material for: A deterministic genotyping workflow reduces waste of transgenic individuals by two-thirds
Source: Sci Rep. 2021 Jul 28;11:15325. doi: 10.1038/s41598-021-94288-0 (PMC8319312; doi:10.1038/s41598-021-94288-0)
Supplement: Supplementary file 1 — Supplementary Figure S1. [file 41598_2021_94288_MOESM1_ESM.docx]

## Figure S1


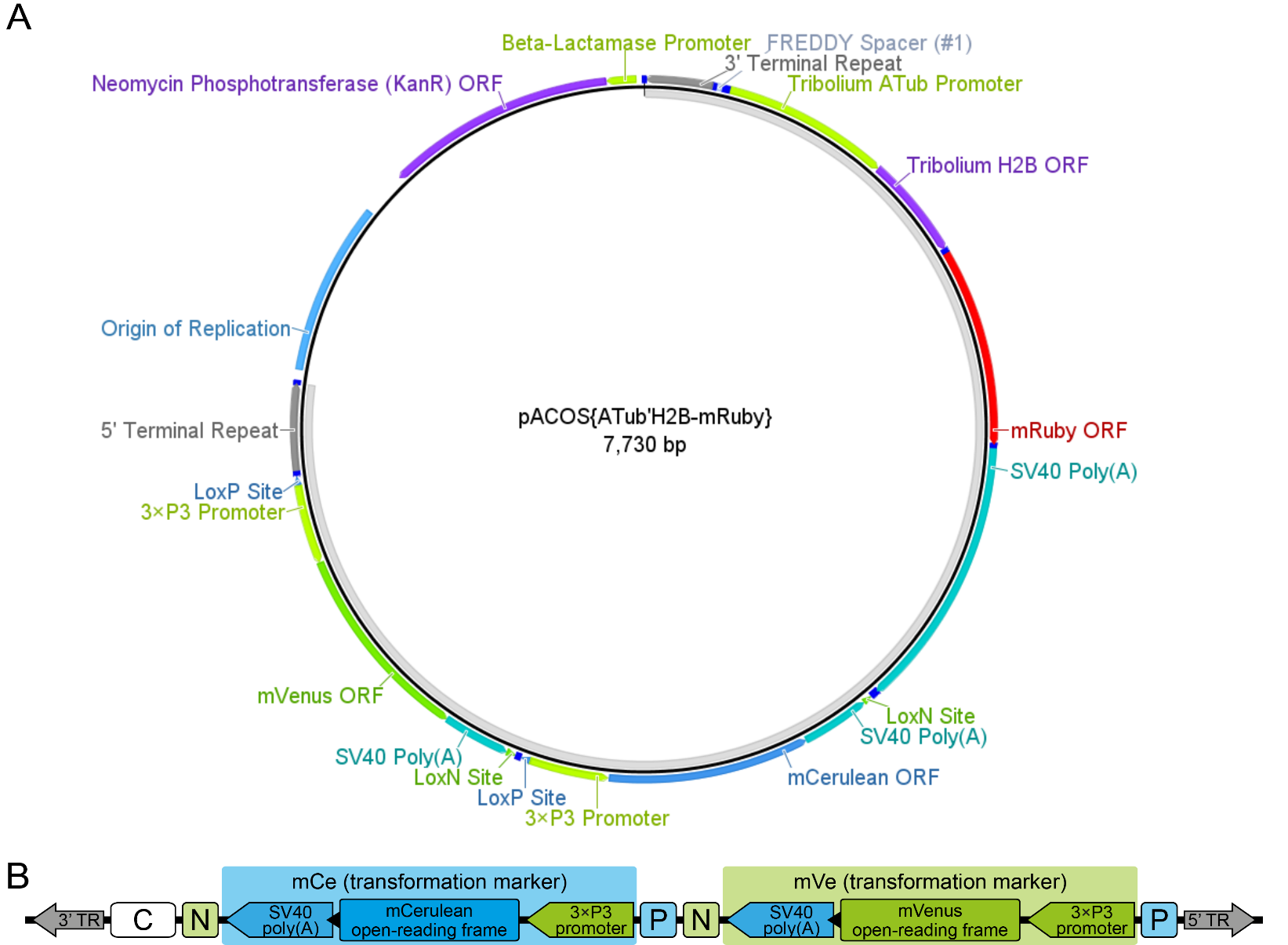


**Figure S1 – The pACOS{ATub’H2B-mRuby} vector. (A)** Vector map of pACOS{ATub’H2B-mRuby}, which is based on pAVOIAF{#1–#2–#3–#4}^6^. In this vector, #1 remains empty, #2 carries an expression cassette for mRuby-labeled *histone H2B* under control of the *tubulin alpha 1-like protein* promoter, while mCe and mVe together with their flanking upstream LoxP and downstream LoxN sites were inserted into #3 and #4, respectively. The unlabeled dark blue boxes represent important restriction enzyme sites. The light gray band on the inside indicates the transgene. Figure created with Geneious 6.1.8 ([www.geneious.com](http://www.geneious.com)). **(B)** Scheme of mCe and mVe, which are embedded into interweaved but incompatible LoxN and LoxP site pairs. Restriction enzyme sites are not shown. Extents of genetic elements are not to scale. ORF, open-reading frame; SV40, simian virus 40; TR, piggyBac terminal repeat; C, cargo (expression cassette described above); N, LoxN site; P, LoxP site.
