# Supplementary material for: A deterministic genotyping workflow reduces waste of transgenic individuals by two-thirds
Source: Sci Rep. 2021 Jul 28;11:15325. doi: 10.1038/s41598-021-94288-0 (PMC8319312; doi:10.1038/s41598-021-94288-0)
Supplement: Supplementary file 2 — Supplementary Figure S2. [file 41598_2021_94288_MOESM2_ESM.docx]

## Figure S2


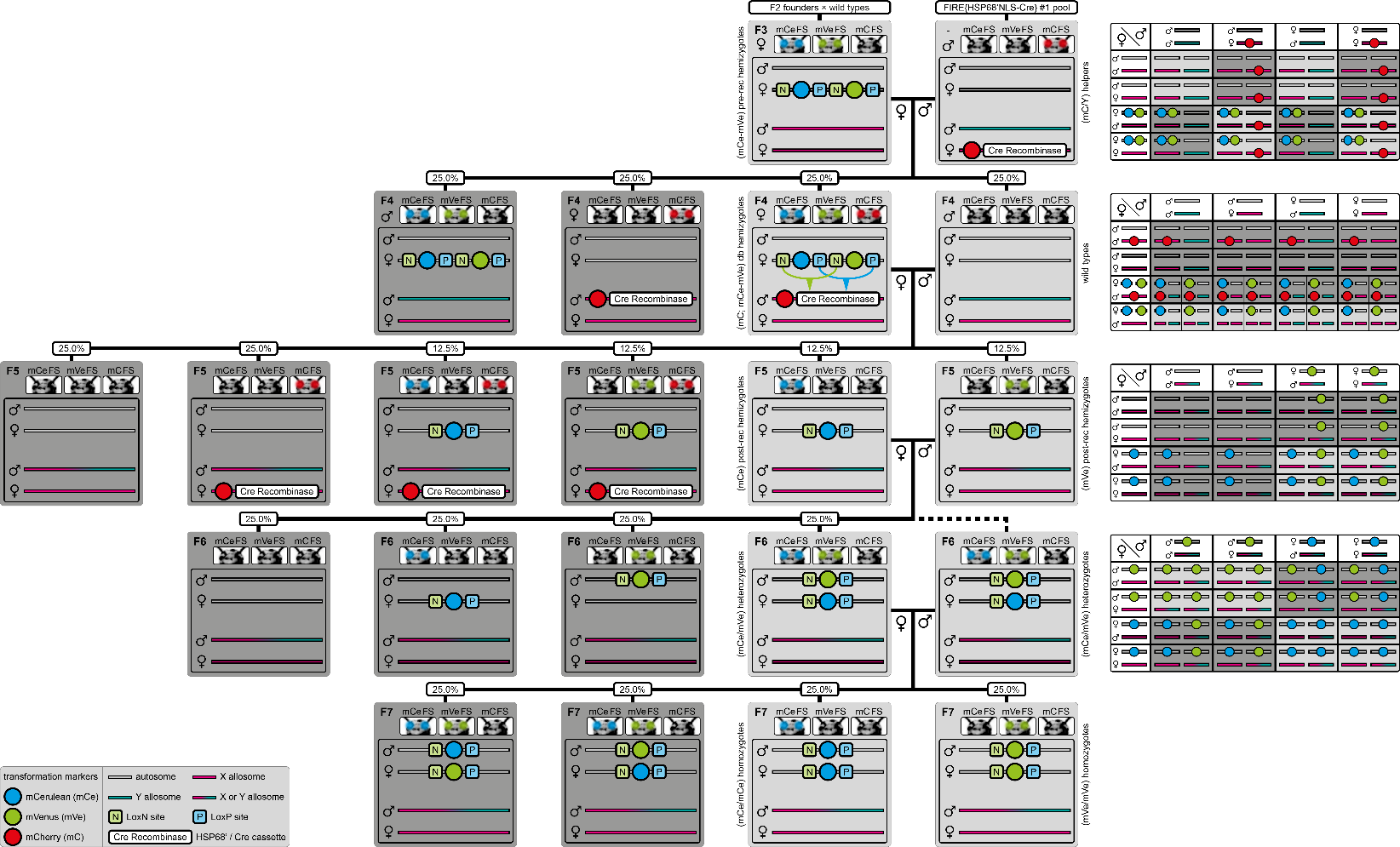


**Figure S2 – The ACOS-associated F3 to F7 mating procedure for the systematic creation of homozygous transgenic lines with all outcomes and respective Punnett squares.** A light gray background indicates either F3 to F6 outcomes that are used further within the procedure or the final F7 (mCe/mCe) and (mVe/mVe) homozygotes. A dark gray background indicates progeny that can be disregarded. Gray bars represent the ACOS transgene location on an autosome pair, pink bars represent the Cre recombinase-expressing helper transgene location on the X allosome and turquoise bars represent the Y allosome. For convenience, the Punnett squares for the F3 to F6 crosses are provided on the right. A F2 (mCe-mVe) founder female × wild-type male cross gives rise to F3 (mCe-mVe) pre-recombination hemizygotes that carry mCe and mVe in cis configuration. A F3 (mCe-mVe) pre-recombination hemizygous female × (mC/Y) FIRE{HSP68’NLS-Cre} #1 helper male cross results in F4 (mC; mCe-mVe) double hemizygotes, in which one marker is removed through Cre-mediated recombination. Next, a F4 (mC; mCe-mVe) double hemizygous female × wild-type male cross gives rise to F5 (mCe) and (mVe) post-recombination hemizygotes. A F5 (mCe) post-recombination hemizygous female × F5 (mVe) post-recombination hemizygous male sibling cross results in F6 (mCe/mVe) heterozygous progeny that carry mCe and mVe in trans configuration. Finally, a F6 (mCe/mVe) heterozygous female × a F6 (mCe/mVe) heterozygous male sibling cross gives rise to F7 (mCe/mCe) and (mVe/mVe) homozygous progeny. The percentage boxes indicate the theoretical ratio of the progeny that carry the respective genotype, the dashed line represents genotypically identical siblings. FS, filter set; rec, recombination; db, double.
