# Supplementary material for: A deterministic genotyping workflow reduces waste of transgenic individuals by two-thirds
Source: Sci Rep. 2021 Jul 28;11:15325. doi: 10.1038/s41598-021-94288-0 (PMC8319312; doi:10.1038/s41598-021-94288-0)
Supplement: Supplementary file 3 — Supplementary Table S1. [file 41598_2021_94288_MOESM3_ESM.docx]

## Table S1

**Table S1 – Mating procedure results for the pACOS{ATub’H2B-mRuby} #1 to #3 sublines from the F3 to the F7 generation.** Progeny that were used in the subsequent crosses or to establish the F7+ continuative cultures are marked in bold. F6-S, F7-mCe and F7-mVe are control crosses. The numbers in brackets and in the ‘total’ sub-column indicate the number of scored individuals.

| **Cross** | **Genotypes** | **Subline** | **Progeny** | | | | | | | | |
| --- | --- | --- | --- | --- | --- | --- | --- | --- | --- | --- | --- |
|  |  |  | 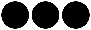 | 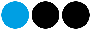 | 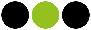 | 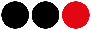 | 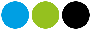 | 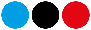 | 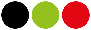 | 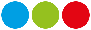 | **total** |
|  |  |  |  |  |  |  |  |  |  |  |  |
| **F3** | 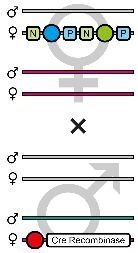 | **Theoretical** | 25.0% | - | - | 25.0% | 25.0% | - | - | **25.0%** | - |
|  |  | **ACOS #1** | 29.8% (23) | - | - | 24.7% (19) | 10.4% (8) | - | - | **35.1% (27)** | 77 |
|  |  | **ACOS #2** | 23.3% (14) | - | - | 26.7% (16) | 25.0% (15) | - | - | **25.0% (15)** | 60 |
|  |  | **ACOS #3** | 32.3% (41) | - | - | 18.1% (23) | 30.7% (39) | - | - | **18.9% (24)** | 127 |
|  |  | **Mean ± SD** | 28.5 ± 4.7% | - | - | 23.2 ± 4.5% | 22.0 ± 10.5% |  |  | **23.0 ± 3.6%** | 88.0 |
|  |  |  |  |  |  |  |  |  |  |  |  |
| **F4** | 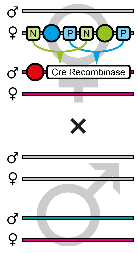 | **Theoretical** | 25.0% | **12.5%** | **12.5%** | 25.0% | - | 12.5% | 12.5% | - | - |
|  |  | **ACOS #1** | 23.0% (54) | **21.3% (50)** | **2.5% (6)** | 31.1% (73) | - | 20.0% (47) | 2.1% (5) | - | 235 |
|  |  | **ACOS #2** | 37.9% (53) | **14.3% (20)** | **3.6% (5)** | 32.1% (45) | - | 6.4% (9) | 5.7% (8) | - | 140 |
|  |  | **ACOS #3** | 26.5% (26) | **19.4% (19)** | **4.1% (4)** | 22.5% (22) | - | 20.4% (20) | 7.1% (7) | - | 98 |
|  |  | **Mean ± SD** | 29.1 ± 7.8% | **18.3 ± 3.6%** | **3.4 ± 0.8%** | 28.6 ± 5.3% | - | 15.6 ± 8.0% | 5.0 ± 2.6% | - | 157.7 |
|  |  |  |  |  |  |  |  |  |  |  |  |
| **F5** | 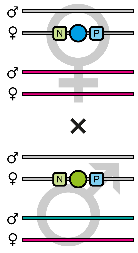 | **Theoretical** | 25.0% | 25.0% | 25.0% | - | **25.0%** | - | - | - | - |
|  |  | **ACOS #1** | 29.7% (19) | 23.4% (15) | 26.6% (17) | - | **20.3% (13)** | - | - | - | 64 |
|  |  | **ACOS #2** | 30.8% (25) | 23.5% (19) | 24.7% (20) | - | **21.0% (17)** | - | - | - | 81 |
|  |  | **ACOS #3** | 24.1% (40) | 21.1% (35) | 23.5% (39) | - | **31.3% (52)** | - | - | - | 166 |
|  |  | **Mean ± SD** | 28.2 ± 3.6% | 22.7 ± 1.4% | 24.9 ± 1.6% | - | **24.2 ± 6.2%** | - | - | - | 103.7 |
|  |  |  |  |  |  |  |  |  |  |  |  |
| **F6-S** | 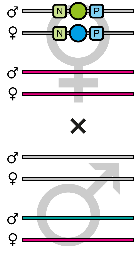 | **Theoretical** | - | 50.0% | 50.0% | - | - | - | - | - | - |
|  |  | **ACOS #1** |  | 51.7% (61) | 48.3% (57) | - | - | - | - | - | 118 |
|  |  | **ACOS #2** | - | 53.7% (29) | 46.3% (25) | - | - | - | - | - | 54 |
|  |  | **ACOS #3** | - | 56.0% (51) | 44.0% (40) | - | - | - | - | - | 91 |
|  |  | **Mean ± SD** | - | 53.8 ± 2.2% | 46.2 ±2.2% | - | - | - | - | - | 87.7 |
|  |  |  |  |  |  |  |  |  |  |  |  |
| **F6** | 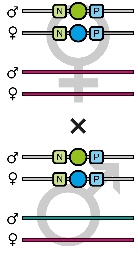 | **Theoretical** | - | **25.0%** | **25.0%** | - | 50.0% | - | - | - | - |
|  |  | **ACOS #1** | - | **30.0% (27)** | **24.4% (22)** | - | 45.6% (41) | - | - | - | 90 |
|  |  | **ACOS #2** | - | **29.4% (42)** | **24.5% (35)** | - | 46.1% (66) | - | - | - | 143 |
|  |  | **ACOS #3** | - | **25.3% (19)** | **18.7% (14)** | - | 56.0% (42) | - | - | - | 75 |
|  |  | **Mean ± SD** | - | **28.2 ± 2.6%** | **22.5 ± 3.3%** | - | 49.2 ± 5.7% | - | - | - | 102.7 |
|  |  |  |  |  |  |  |  |  |  |  |  |
| **F7-mCe** | 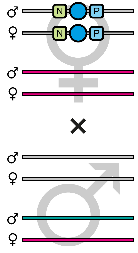 | **Theoretical** | - | 100% | - | - | - | - | - | - | - |
|  |  | **ACOS #1** | - | 100% (45) | - | - | - | - | - | - | 45 |
|  |  | **ACOS #2** | - | 100% (51) | - | - | - | - | - | - | 51 |
|  |  | **ACOS #3** | - | 100% (61) | - | - | - | - | - | - | 61 |
|  |  | **Mean** | - | 100% | - | - | - | - | - | - | 52.3 |
|  |  |  |  |  |  |  |  |  |  |  |  |
| **F7-mVe** | 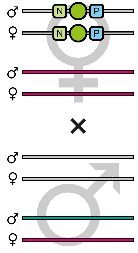 | **Theoretical** | - | - | 100% | - | - | - | - | - | - |
|  |  | **ACOS #1** | - | - | 100% (87) | - | - | - | - | - | 87 |
|  |  | **ACOS #2** | - | - | 100% (80) | - | - | - | - | - | 80 |
|  |  | **ACOS #3** | - | - | 100% (83) | - | - | - | - | - | 83 |
|  |  | **Mean** | - | - | 100% | - | - | - | - | - | 83.3 |
