# Supplementary material for: A deterministic genotyping workflow reduces waste of transgenic individuals by two-thirds
Source: Sci Rep. 2021 Jul 28;11:15325. doi: 10.1038/s41598-021-94288-0 (PMC8319312; doi:10.1038/s41598-021-94288-0)
Supplement: Supplementary file 4 — Supplementary Table S2. [file 41598_2021_94288_MOESM4_ESM.docx]

## Table S2

**Table S2 – F9- and F10-associated control cross results for the Gruul #1 to #3 hybrid sublines.** The numbers in brackets and in the ‘total’ sub-column indicate the number of scored individuals. No significant differences between the arithmetic means and the theoretical Mendelian ratios were found. SD, standard deviation; n.s., not significant.

| **Cross** | **Genotypes** | **Subline** | **Progeny** | | | | | | |
| --- | --- | --- | --- | --- | --- | --- | --- | --- | --- |
|  |  |  | 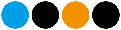 | 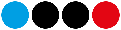 | 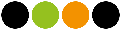 | 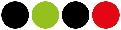 | 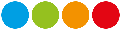 | **other** | **total** |
|  |  |  |  |  |  |  |  |  |  |
| **F9-S** | 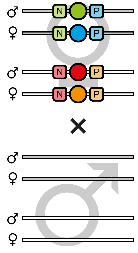 | **Theoretical** | 25.0% | 25.0% | 25.0% | 25.0% | - | - | - |
|  |  | **Gruul #1** | 21.9% (25) | 19.3% (22) | 27.2% (31) | 31.6% (36) | - | - | 114 |
|  |  | **Gruul #2** | 16.9% (14) | 20.5% (17) | 30.1% (25) | 32.5% (83) | - | - | 83 |
|  |  | **Gruul #3** | 31.7% (40) | 31.0% (39) | 26.2% (33) | 11.1% (14) | - | - | 126 |
|  |  | **Mean ± SD** | 23.5 ± 7.5% | 23.6 ± 5.3% | 27.8 ± 2.0% | 25.1 ± 12.1% | - | - | 107.7 |
|  |  | **Significance** | n.s. | n.s. | n.s. | n.s. | - | - | - |
|  |  |  |  |  |  |  |  |  |  |
| **F10-mO-mCe** | 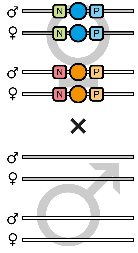 | **Theoretical** | 100% | - | - | - | - | - | - |
|  |  | **Gruul #1** | 100% (122) | - | - | - | - | - | 122 |
|  |  | **Gruul #2** | 100% (106) | - | - | - | - | - | 106 |
|  |  | **Gruul #3** | 100% (104) | - | - | - | - | - | 104 |
|  |  | **Mean** | 100% | - | - | - | - | - | 110.7 |
|  |  | **Significance** | n.s. | - | - | - | - | - | - |
|  |  |  |  |  |  |  |  |  |  |
| **F10-mC-mCe** | 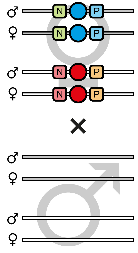 | **Theoretical** | - | 100% | - | - | - | - | - |
|  |  | **Gruul #1** | - | 100% (79) | - | - | - | - | 79 |
|  |  | **Gruul #2** | - | 100% (88) | - | - | - | - | 88 |
|  |  | **Gruul #3** | - | 100% (66) | - | - | - | - | 66 |
|  |  | **Mean** | - | 100% | - | - | - | - | 77.7 |
|  |  | **Significance** | - | n.s. | - | - | - | - | - |
|  |  |  |  |  |  |  |  |  |  |
| **F10-mO-mVe** | 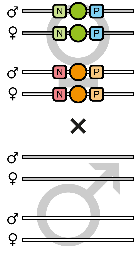 | **Theoretical** | - | - | 100% | - | - | - | - |
|  |  | **Gruul #1** | - | - | 100% (72) | - | - | - | 72 |
|  |  | **Gruul #2** | - | - | 100% (43) | - | - | - | 43 |
|  |  | **Gruul #3** | - | - | 100% (201) | - | - | - | 201 |
|  |  | **Mean** | - | - | 100% | - | - | - | 105.3 |
|  |  | **Significance** | - | - | n.s. | - | - | - | - |
|  |  |  |  |  |  |  |  |  |  |
| **F10-mC-mVe** | 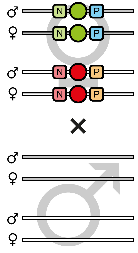 | **Theoretical** | - | - | - | 100% | - | - | - |
|  |  | **Gruul #1** | - | - | - | 100% (81) | - | - | 81 |
|  |  | **Gruul #2** | - | - | - | 100% (98) | - | - | 98 |
|  |  | **Gruul #3** | - | - | - | 100% (53) | - | - | 53 |
|  |  | **Mean** | - | - | - | 100% | - | - | 77.3 |
|  |  | **Significance** | - | - | - | n.s. | . | . | . |
