# Supplementary material for: A deterministic genotyping workflow reduces waste of transgenic individuals by two-thirds
Source: Sci Rep. 2021 Jul 28;11:15325. doi: 10.1038/s41598-021-94288-0 (PMC8319312; doi:10.1038/s41598-021-94288-0)
Supplement: Supplementary file 5 — Supplementary Table S3. [file 41598_2021_94288_MOESM5_ESM.docx]

## Table S3

**Table S3 – F14- and F15-associated control cross results for the Gruul #1 to #3 hybrid sublines.** The numbers in brackets and in the ‘total’ sub-column indicate the number of scored individuals. No significant differences between the arithmetic means and the theoretical Mendelian ratios were found. SD, standard deviation; n.s., not significant.

| **Cross** | **Genotypes** | **Subline** | **Progeny** | | | | | | |
| --- | --- | --- | --- | --- | --- | --- | --- | --- | --- |
|  |  |  | 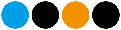 | 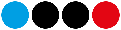 | 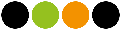 | 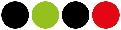 | 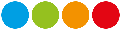 | **other** | **total** |
|  |  |  |  |  |  |  |  |  |  |
| **F14-S** | 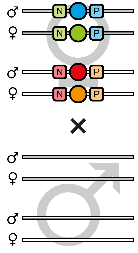 | **Theoretical** | 25.0% | 25.0% | 25.0% | 25.0% | - | - | - |
|  |  | **Gruul #1** | 22.2% (22) | 19.2% (19) | 31.3% (31) | 27.3% (27) | - | - | 99 |
|  |  | **Gruul #2** | 18.2% (20) | 28.2% (31) | 28.2% (31) | 25.4% (28) | - | - | 110 |
|  |  | **Gruul #3** | 20.6% (78) | 23.7% (23) | 26.8% (26) | 28.9% (28) | - | - | 97 |
|  |  | **Mean ± SD** | 20.3 ± 2.0% | 23.7 ± 4.5% | 28.8 ± 2.3% | 27.2 ± 1.8% | - | - | 102.0 |
|  |  | **Significance** | n.s. | n.s. | n.s. | n.s. | - | - | . |
|  |  |  |  |  |  |  |  |  |  |
| **F15-mO-mCe** | 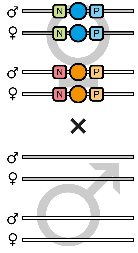 | **Theoretical** | 100% | - | - | - | - | - | - |
|  |  | **Gruul #1** | 100% (91) | - | - | - | - | - | 91 |
|  |  | **Gruul #2** | 100% (56) | - | - | - | - | - | 56 |
|  |  | **Gruul #3** | 100% (78) | - | - | - | - | - | 78 |
|  |  | **Mean** | 100% | - | - | - | - | - | 75.0 |
|  |  | **Significance** | n.s. | - | - | - | - | - | - |
|  |  |  |  |  |  |  |  |  |  |
| **F15-mC-mCe** | 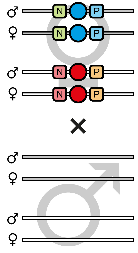 | **Theoretical** | - | 100% | - | - | - | - | - |
|  |  | **Gruul #1** | - | 100% (66) | - | - | - | - | 66 |
|  |  | **Gruul #2** | - | 100% (70) | - | - | - | - | 70 |
|  |  | **Gruul #3** | - | 100% (93) | - | - | - | - | 93 |
|  |  | **Mean** | - | 100% | - | - | - | - | 76.3 |
|  |  | **Significance** | - | n.s. | - | - | - | - | - |
|  |  |  |  |  |  |  |  |  |  |
| **F15-mO-mVe** | 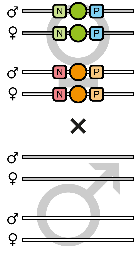 | **Theoretical** | - | - | 100% | - | - | - | - |
|  |  | **Gruul #1** | - | - | 100% (100) | - | - | - | 100 |
|  |  | **Gruul #2** | - | - | 100% (82) | - | - | - | 82 |
|  |  | **Gruul #3** | - | - | 100% (85) | - | - | - | 85 |
|  |  | **Mean** | - | - | 100% | - | - | - | 89.0 |
|  |  | **Significance** | - | - | n.s. | - | - | - | - |
|  |  |  |  |  |  |  |  |  |  |
| **F15-mC-mVe** | 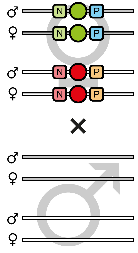 | **Theoretical** | - | - | - | 100% | - | - | - |
|  |  | **Gruul #1** | - | - | - | 100% (73) | - | - | 73 |
|  |  | **Gruul #2** | - | - | - | 100% (76) | - | - | 76 |
|  |  | **Gruul #3** | - | - | - | 100% (93) | - | - | 93 |
|  |  | **Mean** | - | - | - | 100% | - | - | 80.7 |
|  |  | **Significance** | - | - | - | n.s. | - | - | - |
