# Supplementary material for: A deterministic genotyping workflow reduces waste of transgenic individuals by two-thirds
Source: Sci Rep. 2021 Jul 28;11:15325. doi: 10.1038/s41598-021-94288-0 (PMC8319312; doi:10.1038/s41598-021-94288-0)
Supplement: Supplementary file 7 — Supplementary Table S5. [file 41598_2021_94288_MOESM7_ESM.docx]

## Table S5

**Table S5 – Stereo microscope filter sets.** All components were obtained from AHF Analysentechnik, Tübingen, Germany.

| **Filter set** | **Excitation filter** | **Beam splitter** | **Emission filter** | **Comment** |
| --- | --- | --- | --- | --- |
| for mCerulean (mCe FS) | 436/20 ET Bandpass  (F49-436) | 455 nm  (F48-455) | 480/40m ET Bandpass  (F47-480) | Blocks mVenus fluorescence nearly completely |
| for mVenus  (mVe FS) | 535/30 ET Bandpass (F47-535) | 515 nm  (F48-515) | 500/20 ET Bandpass  (F49-500) | Blocks mCerulean and mOrange fluorescence nearly completely |
| for mOrange  (mO FS) | 546/10 ET Bandpass  (F49-547) | 565 nm  (F33-565V20) | 575/15 BrightLine HC  (F39-575) | Blocks mVenus and mCherry fluorescence nearly completely |
| for mCherry  (mC FS) | 586/15 BrightLine HC  (F37-589) | 595 nm  (F43-595V20) | 645/75 ET Bandpass  (F47-645) | Blocks mOrange fluorescence nearly completely |
