# Supplementary material for: A deterministic genotyping workflow reduces waste of transgenic individuals by two-thirds
Source: Sci Rep. 2021 Jul 28;11:15325. doi: 10.1038/s41598-021-94288-0 (PMC8319312; doi:10.1038/s41598-021-94288-0)
Supplement: Supplementary file 10 — Supplementary Legends. [file 41598_2021_94288_MOESM10_ESM.docx]

Movie 1: Long-term fluorescence live imaging of a (mO/mO; mCe/mCe) double homozygous Tribolium embryo from the Gruul #1 hybrid line. Embryogenesis is shown along four directions from 00:00 h to 35:30 h with an interval of 00:30 h between the time points. The movie starts with the rearrangement of the blastoderm and ends during germband retraction. During gastrulation, the ventrally located serosa window is closed by a contracting actomyosin cable that separates the serosa and the amnion. Frame rate is five frames per second. ZA, Z maximum projection with intensity adjustment.
