# Supplementary material for: Author Correction: A deterministic genotyping workflow reduces waste of transgenic individuals by two-thirds
Source: Sci Rep. 2021 Oct 7;11:20335. doi: 10.1038/s41598-021-99823-7 (PMC8497599; doi:10.1038/s41598-021-99823-7)
Supplement: Supplementary file 1 — Supplementary Information. [file 41598_2021_99823_MOESM1_ESM.docx]

## Table S6

| **Dataset (DS)** | **DS0001** | **DS0002** | **DS0003** |
| --- | --- | --- | --- |
| Species | *Tribolium castaneum* (Herbst)  Arthropoda → Insecta → Coleoptera → Tenebrionidae | | |
| Line | AGOC{Zen1’#O(LA)  -mEmerald} #1 ^6^ | ACOS{ATub’H2B-  mRuby} #1 | Gruul #1  hybrid line |
| Line Genotype | one insert  (mC/mC) homozygous | one insert  (mCe/mCe) homozygous | one insert (mO/mO; mCe/mCe)  double homozygous |
| Stock | ~200-500 adults, less than 1 months old | | |
| Stock Medium | full grain wheat flour (113061006, Demeter, Darmstadt, Germany)  supplemented with 5% (wt/wt) inactive dry yeast  (62-106, Flystuff, San Diego, CA, USA) | | |
| Stock Conditions | 12:00 h light / 12:00 h darkness at 25°C and 70% relative humidity  (DR-36VL, Percival Scientific, Perry, IA, USA) | | |
| Egg Laying Period | 01:00 h at 25°C and 70% relative  humidity exposed to light | | |
| Egg Laying Medium | 405 fine wheat flour (113061036, Demeter, Darmstadt, Germany)  supplemented with 5% (wt/wt) inactive dry yeast  (62-106, Flystuff, San Diego, CA, USA) | | |
| Pre-imaging Incubation | 15:00 h at 25°C and 70% relative humidity in darkness, 01:00 h at room temperature (23±1°C) | | |
| LSFM Type | mDSLM (monolithic digital scanned laser light sheet-based fluorescence microscope) based on DSLM^17^ | | |
| Laser Lines | 488 nm / 20 mW diode laser (PhoxX 488-20, Omicron Laserprodukte GmbH, Rodgau-Dudenhofen, Germany)  561 nm / 25 mW DPSSL (Cobolt Jive CW 561,Omicron Laserprodukte GmbH, Rodgau-Dudenhofen, Germany) | | |
| Excitation Objective | 2.5× NA 0.06 EC Epiplan-Neofluar objective (422320-9900-000**,** Carl Zeiss, Göttingen, Germany) | | |
| Emission Objective | 10× NA 0.3 W N-Achroplan objective (420947-9900-000, Carl Zeiss, Göttingen, Germany) | | |
| Emission Filters | 525/50 single-band bandpass filter (FF03-525/50-25, Semrock/AHF Analysentechnik AG, Tübingen, Germany)  607/70 single-band bandpass filter (FF01-607/70-25, Semrock/AHF Analysentechnik AG, Tübingen, Germany) | | |
| Camera | High-resolution CCD (Clara, Andor, Belfast, United Kingdom), 14 bit, 1040×1392 pixel (pitch 6.45 µm) | | |
| Dataset File Type | TIFF, 16 bit grayscale (planes saved as Z stacks in ZIP-compressed container files, indicated as PL(ZS)) | | |
| Dechorionation | ~60-90 s in 10% (vol/vol) sodium hypochlorite (425044-250ML, Sigma Adlrich, Taufkirchen, Germany)  in autoclaved tap water | | |
| Mounting Method | Cobweb holder (embryos are glued to a thin agarose film spanning a slotted hole)^40^ | | |
| Mounting Agarose | 1% (wt/vol) low-melt agarose (6351.2, Carl Roth, Karlsruhe, Germany)  in autoclaved tap water | | |
| Imaging Buffer | autoclaved tap water | | |
| Imaging Temperature | room temperature (23±1°C) | room temperature (23±1°C) | room temperature (23±1°C) |
| Retrieval | developed to healthy adult,  produced fertile progeny | developed to healthy adult,  produced fertile progeny | developed to healthy adult,  produced fertile progeny |

| **Dataset (DS)** | **DS0001** | **DS0002** | **DS0003** |
| --- | --- | --- | --- |
| Dataset Size | 31.4 Gigabyte (TIFF) | 38.9 Gigabyte (TIFF) | 37.9 Gigabyte (TIFF) |
| Figures | - | - | 2 |
| Supplementary Movies | - | - | 1 |
| Comment | - | - | - |
| **Time Points (TP)** | **100 (TP0001-TP0100)** | **145 (TP0001-TP0145)** | **72 (TP0001-TP0072)** |
| TP Interval | 00:30 h | 00:30 h | 00:30 h |
| Total Time (TP×TP Interval) | 49:30 h | 72:00 h | 35:30 h |
| **Directions (DR)** | **4 (DR0001-DR0004)** | **4 (DR0001-DR0004)** | **4 (DR0001-DR0004)** |
| DR Orientations | 0°, 90°, 180°, 270° | 0°, 90°, 180°, 270° | 0°, 90°, 180°, 270° |
| **Channels (CH)** | **1 (CH0001)** | **1 (CH0001)** | **2 (CH0001)** |
| CH0001 Excitation | 488 nm | - | 488 nm |
| CH0001 Power | 135 µW (close to the embryo) | - | 135 µW (close to the embryo) |
| CH0001 Exposure Time | 50 ms | - | 50 ms |
| CH0001 Emission Filter | 525/50 single-band bandpass filter | - | 525/50 single-band bandpass filter |
| CH0002 Excitation | - | 561 nm | 561 nm |
| CH0002 Power | - | 135 µW (close to the embryo) | 135 µW (close to the embryo) |
| CH0002 Exposure Time | - | 50 ms | 50 ms |
| CH0002 Emission Filter | - | 607/70 single-band bandpass filter | 607/70 single-band bandpass filter |
| **Planes (PL)** | **150 (PL0001-PL0150)** | **150 (PL0001-PL0150)** | **150 (PL0001-PL0100)** |
| Z Spacing | 2.58 µm | 2.58 µm | 2.58 µm |
| Z Distance (PL×Z Spacing) | 387.0 µm | 387.0 µm | 387.0 µm |
| **X-Dimensions (XD)** | **600 pixels (cropped)** | **600 pixels (cropped)** | **600 pixels (cropped)** |
| X Spacing | 0.645 µm | 0.645 µm | 0.645 µm |
| X Length (XD×X Spacing) | 387.0 µm | 387.0 µm | 387.0 µm |
| **Y-Dimensions (YD)** | **1000 pixels (cropped)** | **1000 pixels (cropped)** | **1000 pixels (cropped)** |
| Y Spacing | 0.645 µm | 0.645 µm | 0.645 µm |
| Y Length (YD×Y Spacing) | 645.0 µm | 645.0 µm | 645.0 µm |
